# Predictive value of whole-brain CT perfusion combined with ABCD3 score for short-term secondary cerebral infarction after TIA

**DOI:** 10.3389/fneur.2023.1244014

**Published:** 2023-09-07

**Authors:** Shushu Liu, Ting Chen, Wei Wu

**Affiliations:** ^1^Department of Radiology, The Second Affiliated Hospital of Chongqing Medical University, Chongqing, China; ^2^Department of Medical Imaging, People’s Hospital of Fengjie, Chongqing, China

**Keywords:** whole brain, CT perfusion, ABCD3 score, transient ischemic attack, secondary cerebral infarction

## Abstract

**Objective:**

To investigate the predictive value of Whole Brain CT Perfusion (WB-CTP) combined with the ABCD3 score in patients with transient ischemic attack (TIA).

**Methods:**

A total of 336 TIA patients with TIA underwent WB-CTP and ABCD3 score assessment within 48 h of admission. Spearman correlation test was performed to analyze the relationship between the degree of vascular stenosis, relative perfusion values, and ABCD3 score. Logistic regression analysis was used to identify independent risk factors for secondary cerebral infarction. Receiver operating characteristic (ROC) curves were generated to evaluate the predictive value of relative cerebral blood flow (rCBF), degree of vascular stenosis, ABCD3 score, and the WB-CTP-ABCD3 combined model for secondary cerebral infarction after TIA. Calibration curves and H-L tests were used to evaluate the predictive efficacy of the model.

**Results:**

Among the 336 TIA patients, 143 showed abnormal perfusion areas and 146 had responsible vessel stenosis. The degree of vascular stenosis, relative time-to-maximum (rTmax), and relative mean transit time (rMTT) were positively correlated with the ABCD3 score, while rCBF and relative cerebral blood volume (rCBV) were negatively correlated with the ABCD3 score. ROC curve analysis identified a cutoff value of 0.8205 for rCBF, with a sensitivity of 84.10% and specificity of 58.10% for distinguishing the cerebral infarction group from the non-cerebral infarction group. Furthermore, rCBF ≤ 0.8205, degree of vascular stenosis, and ABCD3 score > 6 were identified as independent risk factors for secondary cerebral infarction in TIA patients within 90 days in TIA patients. The AUC of the WB-CTP-ABCD3 combined model for predicting secondary cerebral infarction within 90 days was 0.836. The model risk was assessed by plotting calibration curves. The value of p for the H-L goodness of fit test was 0.366 (*p* > 0.05), which indicated that the difference between the obtained model and the perfect model were statistically insignificant.

**Conclusion:**

The combined model of WB-CTP-ABCD3 shows promise as a valuable method for predicting secondary cerebral infarction within 90 days following TIA.

## Introduction

Transient ischemic attack (TIA) is a common, high-risk clinical syndrome characterized by acute neurological deficits lasting less than 24 h. It serves as a warning sign for stroke events, and approximately 25% of TIA patients experience adverse events such as recurrence, infarction, or death within 90 days. Therefore, early identification of risk factors for secondary cerebral infarction, risk stratification, and personalized treatment are crucial for TIA prognosis (1, 2).

The ABCD score has been widely used as a predictive tool for assessing TIA stroke risk. Among the various ABCD scores, the ABCD3 score has demonstrated better predictive efficacy than the ABCD2 score. The ABCD3-I score incorporates imaging assessments, such as diffusion-weighted imaging (DWI) high signal and ipsilateral carotid artery stenosis ≥50%. It has shown higher predictive value, with a 90-day secondary stroke prediction efficacy of 0.71 (3–5). However, DWI imaging may not accurately reflect the microcirculation status in TIA patients with early-stage cerebral perfusion defects, and it may not be practical for emergency TIA cases (6, 7).

Whole-brain CT perfusion (WB-CTP) imaging is a non-invasive examination that provides valuable information about cerebral hemodynamic changes in the pre-infarction period and evaluates the status of intracranial vessels. WB-CTP imaging can quantitatively evaluate cerebral perfusion abnormalities and vascular stenosis in patients with TIA (8–11). Compared with DWI, WB-CTP imaging overcomes the specific contraindications associated with MRI examinations and allows for immediate assessment, making it more suitable for emergency settings (12–15). However, there are no reports on WB-CTP imaging as an imaging examination combined with ABCD3 score to assess the risk of cerebral infarction secondary to TIA perfusion.

Our hypothesis is that cerebral perfusion values and cerebral vascular status obtained through WB-CTP can serve as risk factors for secondary cerebral infarction in TIA patients within 90 days in patients with TIA. Additionally, we propose that combining these WB-CTP findings with the ABCD3 score can enhance the predictive ability for secondary cerebral infarction in TIA patients within 90 days. This study aims to assess the predictive value of the combined ABCD3 score and WB-CTP in predicting secondary cerebral infarction in patients with TIA within 90 days in TIA patients.

## Methods

### Patients

The study protocol was approved by our institutional review board, and a signed informed consent had been obtained from all participants before enrolling them in this study. According to the TIA diagnostic criteria proposed by American Heart Association/American Stroke Association (16), we collected data from patients diagnosed with TIA between November 2020 and October 2021. Upon admission, all patients underwent an assessment of the ABCD3 score, and WB-CTP was performed within 48 h. The inclusion criteria for the study were as follows: (1) Sudden onset of local symptoms and signs of neurological deficit with complete resolution within 24 h, and (2) Patient cooperation with the examination and negative iodine allergy test. Exclusion criteria included: (1) History in review of cerebral hemorrhage, brain tumor, subdural hematoma, or other cranial or cerebral surgeries; (2) Bilateral carotid or basilar artery stenosis; (3) Severe liver or kidney dysfunction (severe hepatic insufficiency was defined as an ALT value > 2 times the upper limit of normal or an AST value > 2 times the upper limit of normal. Severe renal insufficiency was defined as a creatinine value greater than 1.5 times the upper limit of normal), or severe heart failure, asthma; and (4) Contraindications to MRI examination.

All patients diagnosed with TIA were treated with dual antiplatelet therapy consisting of aspirin (Bayer, 100 mg/day) and clopidogrel (Sanofi, 75 mg/day), with symptoms initiated within 24 h of onset and continued for 21 days (17, 18). Additionally, statins were administered to stabilize plaques and effectively control blood lipid levels, blood glucose levels, and blood pressure during the 90-day follow-up period.

### ABCD3 score

ABCD3 score was calculated according to the following criteria (5): (1) Dual events with ≥1 additional TIA or amaurosis fugax within 7 days of the presenting event were assigned 2 points. (2) Age ≥ 60 years was assigned 1 point. (3) A first recorded blood pressure ≥ 140 systolic or ≥90 diastolic was assigned 1 point. (4) Clinical features of the presenting event with focal weakness were assigned 2 points, while speech impairment without weakness was assigned 1 point. (5) The duration of the presenting event was assigned 1 point for 10–59 min as 1 point, and 2 points for ≥60 min. (6) The presence of diabetes was assigned 1 point. The total score was then calculated by summing the scores of each item was calculated.

### WB-CTP imaging protocol

All patients underwent imaging using a multidetector dual-source Somatom Drive scanner (Siemens Healthineers, Erlangen, Germany). A total of 40 mL of non-ionic contrast agent Iopromide injection (370 mg/mL) was injected through the cubital vein at a flow rate of 5 mL/s. WB-CTP imaging was performed using an adaptive 4D spiral scanning protocol with a total scan time of 40–45 s. The acquired image data were transferred to the image processing workstation (Syngo.Via, version VB10B, Siemens Healthineers, Erlangen, Germany) for automatic analysis of cerebral perfusion. The brain perfusion software generated pseudocolor perfusion images of time-to-maximum (Tmax), mean transit time (MTT), cerebral blood flow (CBF), and cerebral blood volume (CBV). For vascular evaluation, dynamic computed tomography angiography (CTA) was reconstructed using 1.5-mm thin-slice CT arterial phase images, and the best CTA image was selected.

### DWI

All patients were followed up for 90 days from the first TIA. If suspected stroke symptoms occurred, DWI was performed immediately.

### Image analysis

#### CTP

The region of interest (ROI) was manually delineated on the affected side and the corresponding contralateral hemisphere, with the midline serving as the central axis. A total of 90–110 pixels were selected for each ROI. The perfusion parameters of the abnormal cerebral perfusion area and the corresponding contralateral cerebral perfusion area were measured symmetrically, and the mean value was calculated based on three measurements. Relative perfusion values were obtained by dividing the perfusion values of the affected side by the perfusion values of the healthy side, resulting in relative (r) Tmax, rMTT, rCBV, and rCBF values, respectively. If multiple abnormal perfusion areas were detected, the most severe abnormal perfusion area was chosen for evaluation.

#### CTA

In the best CTA images, the method used for determining the percentage of stenosis of intracranial vessels followed the same approach as that used in the Warfarin-Aspirin Symptomatic Intracranial Disease (WASID) study (19). The degree of stenosis was classified as follows: no obvious stenosis, mild stenosis (stenosis degree < 30%), moderate stenosis (stenosis degree 30%–69%), and severe or occlusion (stenosis degree 70%–100%) (20). If there was only one stenosis was observed in the unilateral internal carotid artery, anterior cerebral artery, middle cerebral artery, or posterior cerebral artery, it was considered the responsible vessel. In cases of multiple stenoses, the most severe stenosis was considered the responsible vessel.

#### DWI

Patients with TIA were followed up for DWI within 90 days of onset. If there was no definite high signal shadow was observed on DWI, it was considered normal. If a high focal high signal was present on DWI corresponding to clinical symptoms, it was classified as abnormal, indicating acute stroke.

The imaging data of all enrolled patients were reviewed in a double-blind manner by two experienced neuroradiologists with more than 10 years of experience. The evaluation included the whole brain CTP, the best CTA, and DWI images. In cases of disagreement, the two neuroradiologists reached a consensus through in-depth discussion.

### Statistical analysis

All statistical analyses were performed using IBM SPSS Statistics version 26.0 software (IBM SPSS Inc., Chicago, IL, United States). Quantitative variables were presented as mean ± standard deviation (SD) or median (interquartile range), while qualitative variables were reported as numbers and percentages. The Mann–Whitney test was utilized to compare the cerebral perfusion parameters between the affected and healthy sides. Spearman correlation test was employed to assess the correlation between ABCD3 score, degree of vascular stenosis, and each relative perfusion parameter value. Logistic regression model was applied to analyze the independent factors associated with secondary cerebral infarction in TIA patients within 90 days. Receiver operating characteristic (ROC) curves were constructed, and the area under the curve (AUC) was calculated. The Hosmer-Lemeshow test was used to evaluate the model, and the calibration curve was used to measure the degree of calibration of the model. The DeLong test was utilized to compare the predictive efficacy of each single model and the combined model for cerebral infarction in TIA patients within 90 days. A value of p of less than 0.05 was considered statistically significant.

## Results

### Patient characteristics

**Figure 1:**
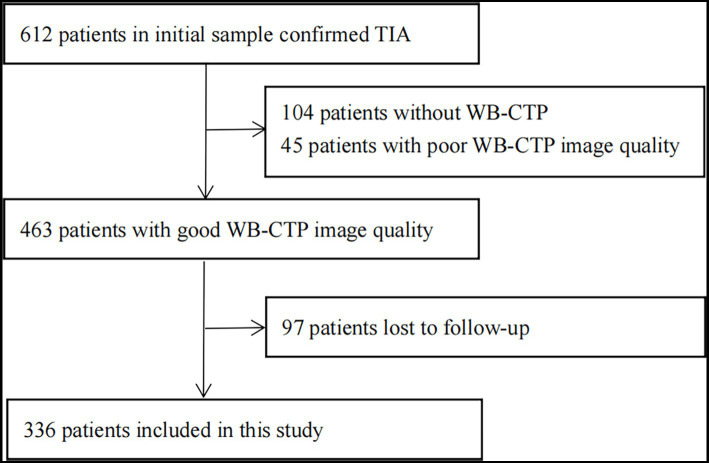
Flow chart of study profile. CT, computed tomography; TIA, transient ischaemic attack; WB-CTP, whole brain CT perfusion.

The flow chart of patient selection is depicted in Figure 1. In total, 336 patients participated in this study. The patients’ characteristics are summarized in Table 1.

**Table 1:** Clinical data and characteristics of 336 TIA patients.

Characteristics	Value
Mean age	62.36 ± 12.12
Male/female	177/159 (52.68%/47.32%)
Coronary heart disease	82 (24.40%)
Hypertension	186 (55.36%)
Diabetes	86 (25.60%)
Dyslipidemia	77 (22.92%)
Dizziness and headache	73 (21.73%)
Paroxysmal unilateral limb weakness	67 (19.94%)
Lalopathy	54 (16.07%)
Transient amaurosis or blindness	50 (14.88%)
Nausea and vomiting	28 (8.33%)
Dysequilibrium	16 (4.76%)
Diplopia	10 (2.98%)

### WB-CTP

A total of 286 perfusion abnormalities were identified in 336 patients with TIA. These abnormalities were distributed as follows: 122 in the frontal lobe, 12 in the temporal lobe, 32 in the parietal lobe, 34 in the occipital lobe, 50 in the basal ganglia, 6 in the thalamus, 14 in the periventricular region, 6 in the centrum semiovale, 2 in the brain stem, and 8 in the cerebellar hemisphere. The comparison results of Tmax, MTT, CBF, and CBV values between the affected and healthy sides are presented in Table 2.

**Table 2:** Comparison of CT perfusion imaging parameters between the affected side and the healthy side in 143 abnormal perfusion areas [P50 (P25, P75)].

Group	CBF (mL/100 mL.min)	MTT(s)	Tmax (s)	CBV (mL/100 g)
Affected side	42.97 (37.61, 50.99)	3.47 (2.99, 4.01)	2.84 (2.20, 3.47)	2.87 (2.21, 3.38)
Healthy side	56.15 (45.67, 65.64)	3.22 (2.48, 3.90)	2.16 (1.73, 2.75)	2.67 (2.09, 3.35)
Wilcoxon Z	−14.087	−9.009	−12.305	−0.047
*p*	*p* < 0.001	*p* < 0.001	*p* < 0.001	0.963

CBF, cerebral blood flow; CBV, cerebral blood volume; MTT, mean transit time; Tmax, time to maximum.

In the 336 patients with TIA, a total of 278 responsible blood vessels were identified. These vessels were categorized as follows: 58 showed no obvious stenosis, 82 had mild stenosis, 112 had moderate stenosis, and 84 had severe stenosis or occlusion. Among these vessels were 91 cases of internal carotid artery stenosis, 60 cases of anterior cerebral artery stenosis, 81 cases of middle cerebral artery stenosis, 36 cases of posterior cerebral artery stenosis, and 10 cases of vertebrobasilar artery stenosis.

### Analysis of correlation

The correlation analyses between the ABCD3 score, the degree of vascular stenosis, and the values of each relative perfusion parameter are shown in Table 3.

**Table 3:** Correlation of ABCD3 score with the degree of vascular stenosis and the values of each relative perfusion parameter.

		Vascular stenosis on CTA	Each relative CTP parameter			
			rCBF	rMTT	rTmax	rCBV
ABCD3 score	r^a^	0.820	−0.465	0.196	0.189	−0.177
	*p-*value	<0.001^b^	<0.001^b^	<0.001^b^	<0.001^b^	0.001^b^

^a^r represents Spearman’s correlation coefficient, with positive values representing positive correlation and negative values representing negative correlation.

^b^There was a significant difference between the two examination methods [value of *p* or adjusted value of *p* (for pairwise comparison) less than 0.05 was statistically significant].

^b^There was a significant difference between the two examination methods [value of *p* or adjusted value of *p* (for pairwise comparison) less than 0.05 was statistically significant]. CT, computed tomography; CTA, CT angiography; CTP, CT perfusion; rCBF, relative cerebral blood flow; rCBV, relative cerebral blood volume; rMTT, relative mean transit time; rTmax, relative time to maximum.

#### Univariate analysis

**Figure 2:**
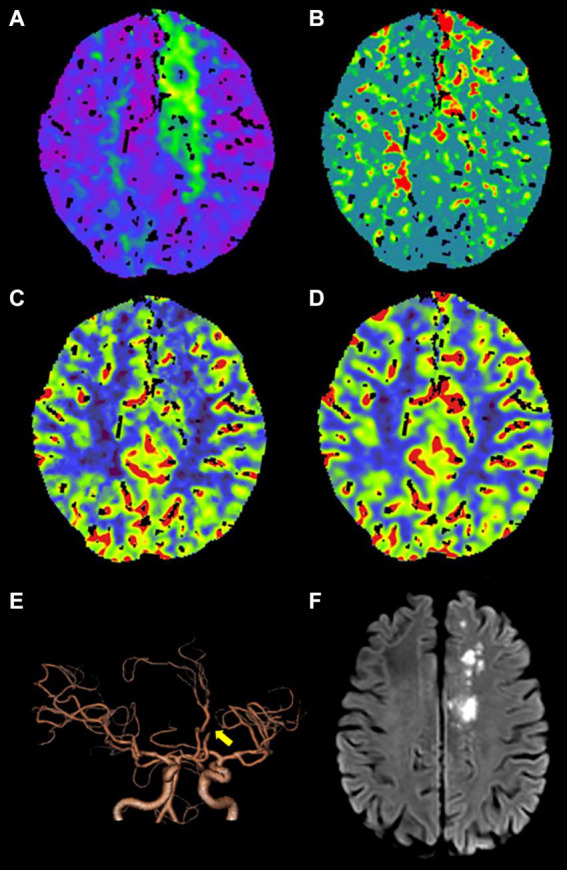
An adult patient with TIA presented with secondary cerebral infarction within 90 days of follow-up. **(A–D)** CTP maps, including time-to-maximum (Tmax), mean transit time (MTT), cerebral blood flow (CBF), and cerebral blood volume (CBV), reveal that Tmax is prolonged **(A)**, MTT is prolonged **(B)**, CBF is slightly decreased **(C)**, and CBV exhibits no obvious change **(D)** in the left frontal lobe and centrum semiovale. **(E)** Computed tomography angiography (CTA) indicates occlusion of A2 segment of the left anterior cerebral artery (yellow arrow). **(F)** DWI shows the secondary cerebral infarction in the left frontal lobe and centrum semiovale within 90 days of follow-up.

**Figure 3:**
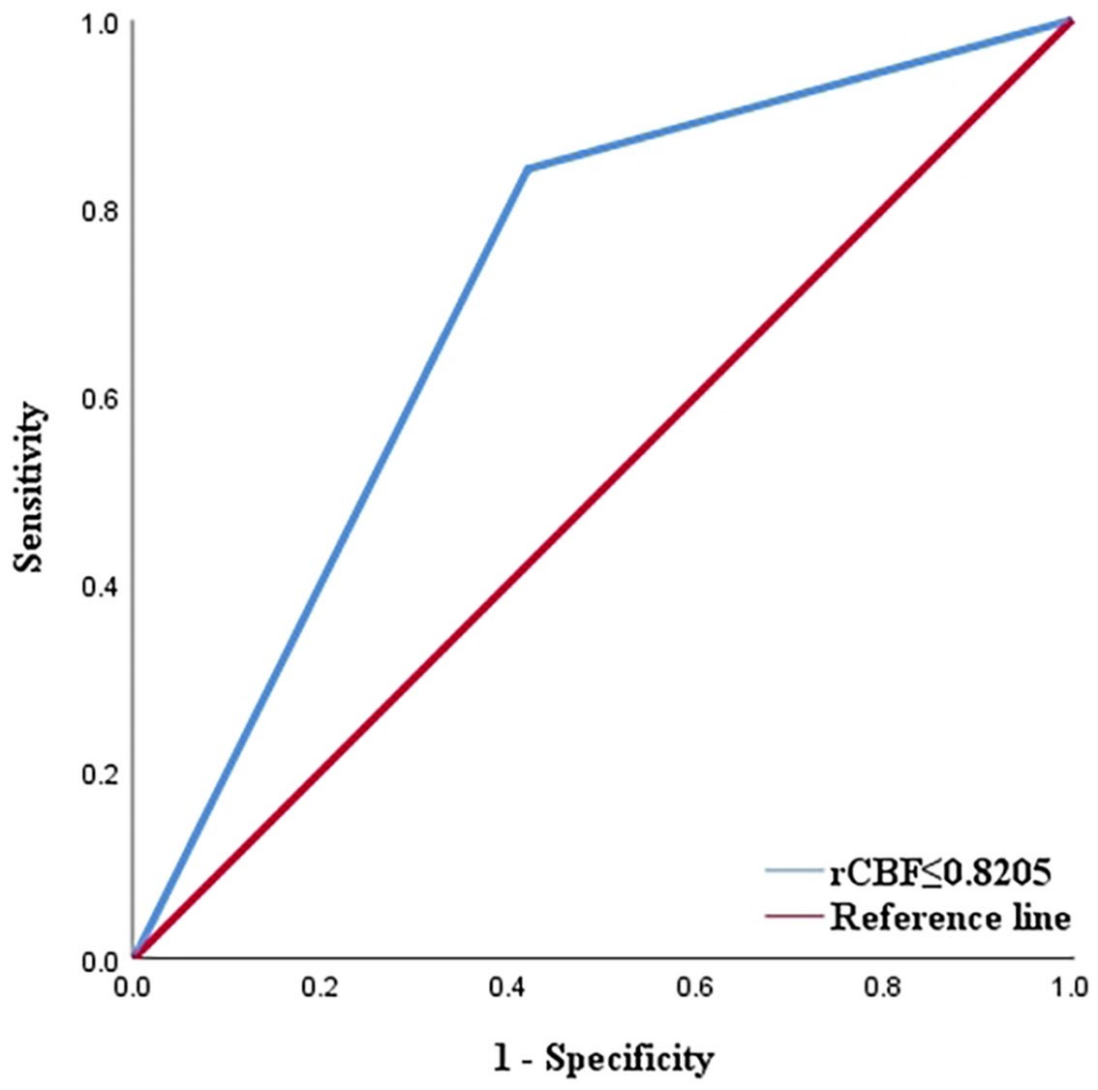
ROC curve displaying the predictive efficacy of rCBF for cerebral infarction in TIA patients ([AUC] = 0.745). AUC, area under curve; ROC, receiver operating characteristic; rCBF, relative cerebral blood flow; TIA, transient ischaemic attack.

Out of the 336 TIA patients, 88 were classified in the infarction group, while 248 were in the non-infarction group during the 90-day follow-up period (Figure 2). The results of the univariate analysis comparing the two groups are presented in Table 4. The ROC curve analysis revealed that the area under the curve (AUC) for rCBF in predicting cerebral infarction in TIA patients at 90 days was 0.745 (Figure 3). The optimal cutoff value for rCBF to predict secondary cerebral infarction in TIA patients at 90 days was determined to be 0.8205, with a sensitivity of 84.10% and a specificity of 58.10%.

**Table 4:** Univariate analysis of factors influencing secondary cerebral infarction in TIA patients within 90 days.

	Infarction group (*n* = 88)	Non-infarction group (*n* = 248)	value of *p*
Age > 60, number (%)	70 (79.55)	134 (54.03)	<0.001
Male, number (%)	53 (60.23)	124 (50.00)	0.099
Coronary heart disease, number (%)	32 (36.36)	50 (20.16)	0.002
Hypertension, number (%)	66 (75.00)	120 (48.39)	<0.001
Diabetes, number (%)	28 (31.82)	58 (23.39)	0.119
Dyslipidemia, number (%)	21 (23.86)	56 (22.58)	0.806
ABCD3 score > 6, number (%)	38 (43.18)	25 (10.08)	<0.001
Degree of vascular stenosis, number (%)			<0.001
No significant stenosis	1 (1.14)	57 (22.98)	
Mild stenosis	10 (11.36)	72 (29.03)	
Moderate stenosis	25 (28.41)	87 (35.08)	
Severe stenosis or occlusion	52 (59.09)	32 (12.90)	
**Values of perfusion parameters [median (interquartile range)]**			
rCBF	0.71 (0.23)	0.84 (0.21)	<0.001
rCBV	0.98 (0.40)	1.02 (0.25)	0.685
rTmax	1.23 (0.75)	1.20 (0.37)	0.161
rMTT	1.07 (0.24)	1.07 (0.18)	0.443

rCBF, relative cerebral blood flow; rCBV, relative cerebral blood volume; rTmax, relative time to maximum; rMTT, relative mean transit time.

#### Multivariate analysis

A logistic regression model was utilized to analyze the occurrence of secondary cerebral infarction occurred within 90 days as the dependent variable. The independent variables included age > 60 years, history of hypertension, coronary heart disease, ABCD3 score > 6 points, degree of vascular stenosis, and rCBF ≤ 0.8205 as the independent variables. The results demonstrated that ABCD3 score > 6, degree of vascular stenosis, and rCBF ≤ 0.8205 were independent risk factors for cerebral infarction in TIA patients within the 90-day follow-up period, with statistical significance (*p* < 0.05; Table 5).

**Table 5:** Multivariate logistic regression analysis of influencing factors for secondary cerebral infarction in TIA patients within 90 days.

	B	Standard error	Wald	Significance	OR (95%CI)
Age > 60	0.000	0.394	0.000	1.000	1.000 (0.461, 2.166)
Hypertension	0.650	0.356	3.326	0.068	1.916 (0.953, 3.852)
Coronary heart disease	0.245	0.345	0.501	0.479	1.277 (0.649, 2.513)
ABCD3 score > 6	0.797	0.399	3.986	0.046	2.218 (1.015, 4.849)
Degree of vascular stenosis	0.848	0.226	14.037	0.000	2.336 (1.499, 3.640)
rCBF ≤ 0.8205	1.438	0.355	16.385	0.000	4.212 (2.099, 8.449)

CI, confidence interval; OR, odds ratio; rCBF, relative cerebral blood flow.

### Predictive efficacy

**Figure 4:**
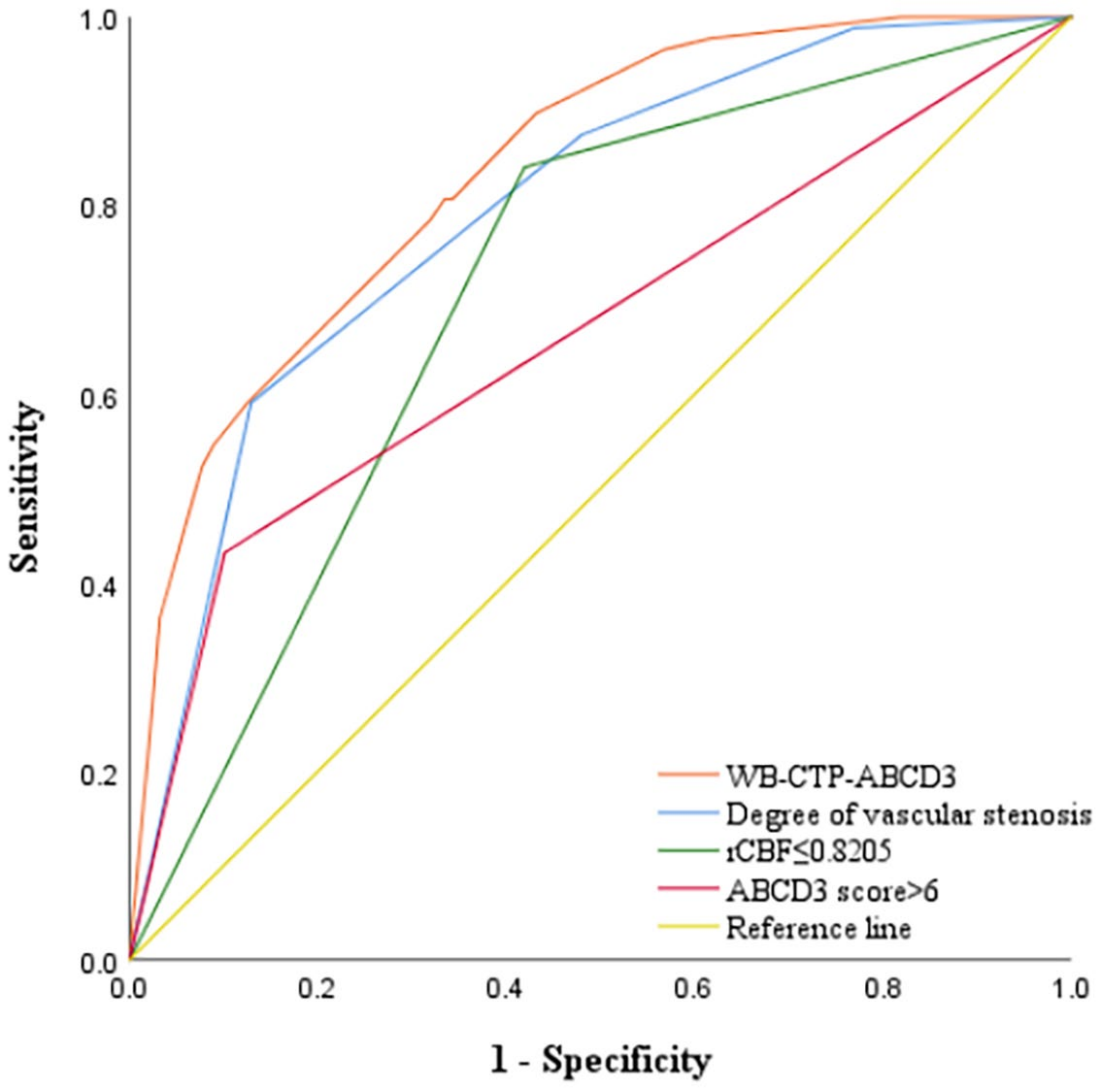
ROC curve depicting the predictive efficacy of each model for secondary cerebral infarction within 90 days. ROC, receiver operating characteristic; rCBF, relative cerebral blood flow; WB-CTP, whole brain CT perfusion.

**Figure 5:**
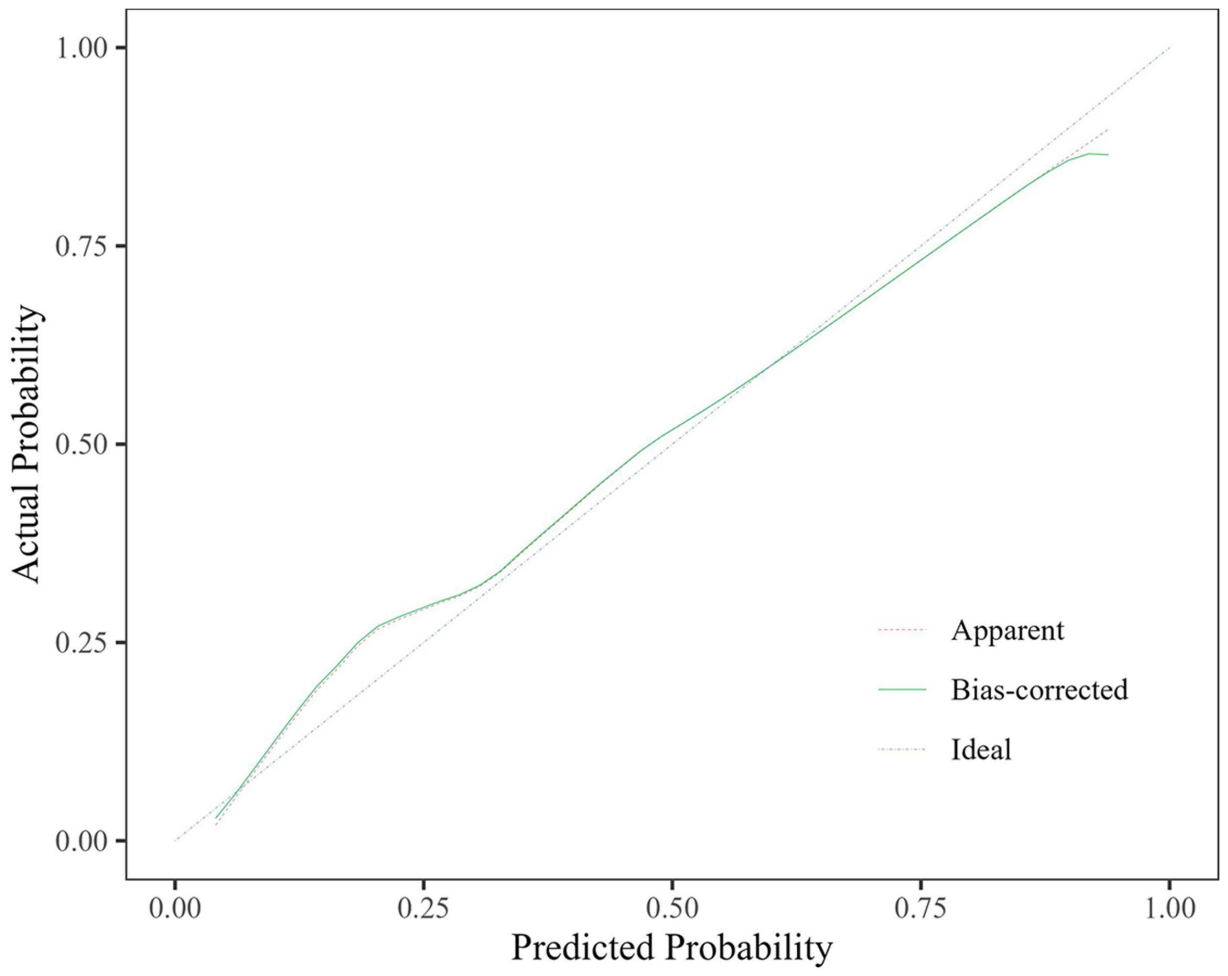
Calibration curve.

The predictive efficacy, sensitivity and specificity of rCBF ≤ 0.8205, ABCD3 > 6 points, degree of vascular stenosis, rCBF ≤ 0.8205 + ABCD3 > 6 points + degree of vascular stenosis (WB-CTP-ABCD3) are shown in Figure 4 and Table 6. The value of p for the H-L goodness of fit test was 0.366 (*p* > 0.05), and the model calibration curve was close to the ideal model, as shown in Figure 5. DeLong analysis showed that the combined model of WB-CTP-ABCD3 was significantly different from the area under the curve of rCBF ≤ 0.8205, ABCD3 > 6, and the degree of vascular stenosis, respectively (*p* < 0.05).

**Table 6:** The predictive efficacy of each model for secondary cerebral infarction within 90 days.

	AUC	95% *CI*	Sensitivity	Specificity
ABCD3 score > 6	0.666	0.594–0.737	43.20%	89.90%
rCBF ≤ 0.8205	0.711	0.651–0.770	84.10%	58.10%
Degree of vascular stenosis	0.794	0.741–0.847	59.10%	87.10%
WB-CTP-ABCD3	0.836	0.790–0.882	80.70%	66.50%

AUC, area under the curve; CI, confidence interval; rCBF, relative cerebral blood flow; WB-CTP, whole brain CT perfusion.

## Discussion

In this study, we utilized whole-brain perfusion pseudo-color maps and whole-brain vascular maps obtained through WB-CTP were used to evaluate TIA patients comprehensively and rapidly (21). Our findings revealed a correlation between the ABCD3 score, degree of intracranial vascular stenosis, and the perfusion parameters in the abnormal brain tissue perfusion areas in TIA patients. We determined that an rCBF value of 0.8205 is the cut off for predicting secondary cerebral infarction within 90 days. The predictive efficacy of WB-CTP-ABCD3 prediction model demonstrated a predictive efficacy of 0.836, which could effectively identifying high-risk TIA patients and aiding in accurate risk stratification.

Among the 336 TIA patients in our study, 286 cases exhibited abnormal perfusion areas, characterized by prolonged Tmax and MTT, decreased CBF, and no significant difference in CBV between the affected and healthy sides. These findings align with the hemodynamic changes observed prior to TIA infarction (6) and are consistent with previous reports. Additionally, the majority of TIA patients exhibited varying degrees of vascular stenosis or occlusion (22). Analysis of the best CTA images obtained through WB-CTP examination in these patients identified 278 responsible vessels. Correlation analysis revealed a positive association between the degree of vascular stenosis, relative perfusion parameters and the ABCD3 score, indicating that more severe the vascular stenosis is associated with higher ABCD3 scores, consistent with previous studies (23).

Regarding perfusion parameters, rMTT and rTmax demonstrated positive correlations with the ABCD3 score, while rCBF and rCBV exhibited negative correlations. Notably, the correlation coefficient r value between rCBF and the ABCD3 score was the highest (r = 0.465). These findings suggest that the degree of cerebral perfusion reduction in TIA patients is closely related to the risk indicated by the ABCD3 score. In cases of cerebral vascular stenosis, initial brain tissue ischemia can be compensated through arteriolar and capillary smooth muscle expansion and collateral circulation. However, as rCBF decreases or worsens, vascular expansion reaches its limit, leading to local microcirculation decompensation occurs, and aggravated brain cell ischemic damage. This ultimately results in irreversible brain tissue damage and eventually leads to cerebral infarction (24). Including “double TIA” in the ABCD3 score allows for assessing the decrease degree of rCBF decrease over a short period of time based on clinical symptoms. Higher ABCD3 scores correspond to greater decreases in rCBF within a short period of time frame, indicating more frequent TIA clinical symptoms (25). The degree of vascular stenosis and perfusion parameters obtained through WB-CTP exhibited correlations with the ABCD3 score, highlighting the WB-CTP has clinical value of WB-CTP in predicting TIA risk similar to ABCD3 scores.

The decrease in cerebral blood flow (CBF) is a fundamental factor in the basis of pathological changes of ischemic cerebrovascular diseases (9), and CBF has a sensitivity of up to 89% in detecting early cerebral ischemia (26). Previous reports have shown that reduced CBF has the strongest correlation with neurological dysfunction in TIA patients at a 90-day follow-up, compared to increased time-to-peak (TTP) or mean transit time (MTT) and reduced cerebral blood volume (CBV) (25). In the univariate analysis of TIA patients’ whole-brain CTP perfusion parameters and secondary cerebral infarction within 90 days in our study, rCBF was found to be associated with secondary cerebral infarction, while rTmax, rMTT, and rCBV were not. The rCBF ≤ 0.8205 is an independent predictor of secondary cerebral infarction within 90 days, with a sensitivity of 84.10% and a specificity of 58.10%. This suggests that TIA patients with a decrease in cerebral blood flow to a ratio of CBF of the affected side to the healthy side ≤ 0.8205 have a high risk of secondary irreversible cerebral infarction within 90 days, emphasizing the necessary for early intervention performed to prevent secondary cerebral infarction. Although previous studies have applied rCBF for infarct core identification, hemorrhagic transformation risk assessment, and neurological outcome prediction (27, 28), but few studies have reported on quantitative analysis of the WB-CTP perfusion parameter rCBF for TIA risk assessment.

Foschi et al. (29) found that an ABCD3 score > 6 is a predictor of TIA secondary stroke, and the risk of TIA progressing to cerebral infarction in a short term increases with an increasing ABCD3 score. In our study, an ABCD3 score > 6 was used as the cut off value, and we found that an ABCD3 score > 6, rCBF ≤ 0.8205, and the degree of vascular stenosis were all independent risk factors for the progression to cerebral infarction within 90 days of TIA. We combined these three factors to construct a combined prediction model, WB-CTP-ABCD3. The AUC of WB-CTP-ABCD3 was 0.836, with a sensitivity of 80.70% and specificity of 66.50%, whereas the AUCs for ABCD3 score > 6, rCBF ≤ 0.8205, and vascular stenosis degree were 0.666, 0.711, and 0.794, respectively. This indicates that the predictive value of WB-CTP-ABCD3 model has a higher predictive value (all *p* < 0.05 by DeLong analysis) compared to individual factors. The ABCD3 score, based on clinical history and signs, is easily affected by subjective factors. However, the application of ABCD3 score combined with WB-CTP objective evaluation improves the predictive value of the ABCD3 score.

Our study has several limitations. Firstly, it is a single-center study with a relative small sample, which might bring several unavoidable data bias in our study. Secondly, we only evaluated intracranial vessels, and the status of extracranial vessels was not included in the study. Thirdly, we only analyzed factors for secondary cerebral infarction within 90 days in TIA patients. The prediction of secondary cerebral infarction in TIA patients in the medium and long term requires further exploration.

## Conclusion

The combination of rCBF ≤ 0.8205, an ABCD3 score > 6, and the degree of vascular stenosis can effectively predict the risk of secondary cerebral infarction in TIA patients within 90 days. The WB-CTP-ABCD3 combined model has the potential value as an effective tool for short-term risk prediction in TIA patients, providing a reliable basis for the early identification of high-risk individuals, stratified management, personalized diagnosis and treatment approaches.

## Data availability statement

The original contributions presented in the study are included in the article/supplementary material, further inquiries can be directed to the corresponding author.

## Ethics statement

The studies involving humans were approved by Ethics Committee of the Second Affiliated Hospital of Chongqing Medical University. The studies were conducted in accordance with the local legislation and institutional requirements. The participants provided their written informed consent to participate in this study. Written informed consent was obtained from the individual (s) for the publication of any potentially identifiable images or data included in this article.

## Author contributions

TC designed the study. WW created and tuned the imaging protocol. SL contributed to data collection. TC and SL drafted the manuscript. All authors contributed to the article and approved the submitted version.

## Conflict of interest

The authors declare that the research was conducted in the absence of any commercial or financial relationships that could be construed as a potential conflict of interest.

## Publisher’s note

All claims expressed in this article are solely those of the authors and do not necessarily represent those of their affiliated organizations, or those of the publisher, the editors and the reviewers. Any product that may be evaluated in this article, or claim that may be made by its manufacturer, is not guaranteed or endorsed by the publisher.
